# The Asian plethodontid salamander preserves historical genetic imprints of recent northern expansion

**DOI:** 10.1038/s41598-021-88238-z

**Published:** 2021-04-28

**Authors:** Jong Yoon Jeon, Ji-hwa Jung, Ho Young Suk, Hang Lee, Mi-Sook Min

**Affiliations:** 1 Research Institute for Veterinary Science and Conservation Genome Resource Bank for Korean Wildlife, College of Veterinary Medicine, Seoul National University, Seoul, 08826 South Korea; 2 Department of Forest Sciences, College of Agriculture and Life Sciences, Seoul National University, Seoul, 08826 South Korea; 3 Department of Life Sciences, Yeungnam University, Gyeongsan, Gyeongsangbuk-do 38541 South Korea

**Keywords:** Evolutionary genetics, Population genetics, Genetic markers, Population genetics, Biogeography, Ecological genetics, Molecular ecology

## Abstract

The Korean Peninsula, located at the southern tip of Northeast Asia, has never been covered by ice sheets and was a temperate refugium during the Pleistocene. *Karsenia koreana*, the sole Asian plethodontid salamander species, occurs only on the southern half of the Korean Peninsula and is thought to have found various climatic refugia. Despite its phylogenetic and biogeographic importance, no population-level genetic analysis has been performed on this species. Here we study the population genetic structure of *K. koreana* using mitochondrial and microsatellite loci to understand the recent historical dispersion process that shaped its current distribution. Overall, the genetic distance between populations correlated well with the spatial distance, and the genetic structure among populations showed signs of a unilateral northward expansion from a southernmost refugium population. Given the distinct genetic structure formed among the populations, the level of historical gene flow among populations appears to have been very low. As the estimated effective population size of *K. koreana* was also small, these results suggest that the small, restricted populations of *K. koreana* are extremely vulnerable to environmental changes that may require high levels of genetic diversity to cope with. Thus, special management strategies are needed to preserve these remnant populations.

## Introduction

Within a geographic area, the distribution of a species reflects its historical dispersion and isolation in the context of geological change and Quaternary climate fluctuation^1,2^. To avoid the advances of Pleistocene ice sheets, many terrestrial animals extirpated or migrated, with some in the northern hemisphere dispersing to the south and occupying sites that served as refugia^3^. Even after the retraction of the ice sheets, numerous species remained in place, further adapting and diversifying, resulting in some southern regions exhibiting high species diversity and endemism^4,5^. Phylogeographic approaches can be used to reconstruct the historical migration routes and diversification processes that species or other higher taxa underwent following glacial period^6,7^.

The Korean Peninsula has features that make it attractive for phylogeographic studies. It is located at the southern tip of Northeast Asia and has never been covered by ice sheets, although it was indirectly affected by the northern glaciation of the Quaternary^8^. This area is characterized by a mountainous terrain resulting from complex geological activity, which offered temperate habitats or glacial refugia during the Quaternary glaciations^9^. Perhaps this is why the Korean Peninsula, despite its relatively small area, is a region with a reasonably high species diversity and endemism. This peninsula is home to ten species of salamander, of which eight are endemic^10,11^. Genetic studies of *Hynobius* and *Onychodactylus* salamander species on the Korean Peninsula detected a high level of intraspecific phylogeographic structure^12,13^, the existence of cryptic diversity^11,12^ and evolutionary relatedness to species inhabiting areas north of the peninsula. In total, these results suggest that the Korean Peninsula provided glacial refugia for salamander species that migrated southward.

One other monotypic genus of salamander is found on the Korean Peninsula—the Korean crevice salamander (*Karsenia koreana*). *Karsenia korena* is the only plethodontid species in Asia, and until this species was first discovered in Korea in 2005^14^, plethodontid salamanders were thought to be native only to Europe and the Americas. There have been various attempts to elucidate the biogeographic and phylogenetic histories of plethodontid salamanders, the most diverse family in the order Urodela^15–19^. However, the existence of one plethodontid species in Northeast Asia has introduced a complication in the reconstruction of the historical dispersion process of Plethodontidae^20^. Since *K. koreana* was first described, researchers have attempted to determine its phylogenetic placement as a means of inferring how this species became distributed in Asia^20,21^. The most convincing hypothesis suggests that a small ancestral group of *K. koreana* migrated from western North America to Eurasia through the Bering Land Bridge around 65 Ma^19,22^.

**Figure 1:**
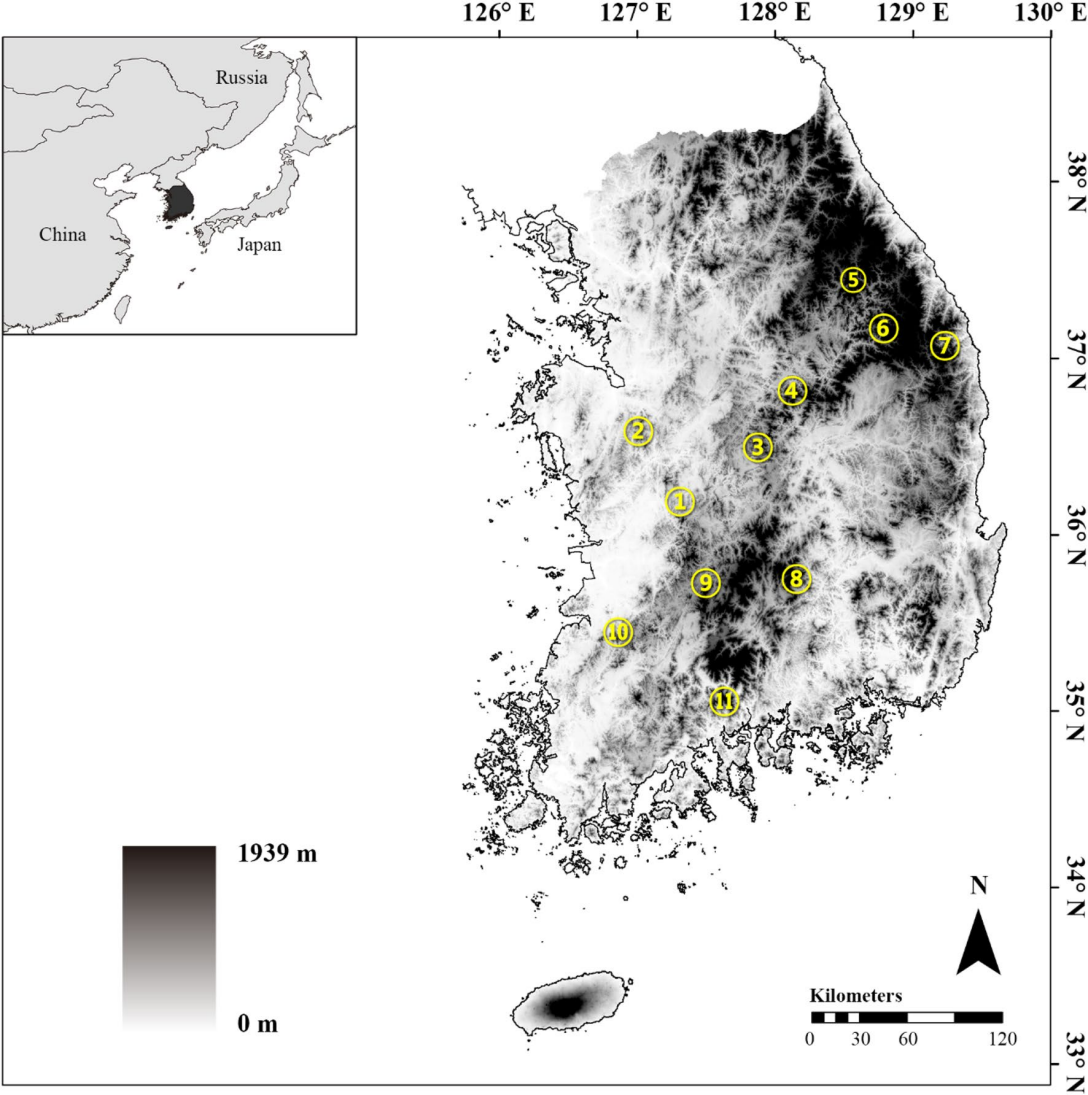
The 11 populations from geographically separate localities of *Karsenia koreana* throughout the Korean Peninsula in this study; the foundational Digital Elevation Model (DEM) file was obtained from http://www.biz-gis.com. Altitude is indicated by the relative darkness, with black being higher elevations. Sites designated as numbers on the map represent the following populations: 1: DaeJeon (DJ), 2: GongJu (GJ), 3: BoEun (BE), 4: JeCheon (JC), 5: PyeongChang (PC), 6: JeongSeon (JS), 7: SamCheock (SC), 8: HapCheon (HC), 9: JinAn (JA), 10: JeongEup (JE) and 11: GwangYang (GY). For the detailed sampling information, see Table 1.

Although officially considered an endangered species^23^, *K. koreana* is widely distributed in and around the mountainous regions of the Korean Peninsula^24^ (Fig. 1). Various aspects of *Karsenia koreana* biology have been studied including its cytogenetics^25^, morphology^26,27^ and ecology^28–30^. However, it is not yet known what recent biogeographic pathways this species has taken to form its current, restricted distribution within the Korean Peninsula. Given that no plethodontid species has been found elsewhere in Asia^10^, the populations from which this species originated (in Russia or northeastern China) have likely been extirpated. The populations on the Korean Peninsula may be considered remnants at the southern end of the biogeographic dispersion. The most effective way to identify the biogeographic pathways of *K. koreana* is to reconstruct the historical patterns of gene flow by measuring the genetic structure and diversity throughout its known distribution. To date, no population-level genetic analysis has been performed on this species.

This study was designed to uncover the population genetic structure of *K. koreana* on the Korean Peninsula to understand the historical dispersion process shaping the structure. Two types of genetic markers were used. First, mitochondrial COI (cytochrome *c* oxidase I) and Cyt *b* (cytochrome *b*) were used to estimate the historical migration and isolation processes of *K. koreana* populations. Second, microsatellites were used to determine the level of gene flow among populations and to estimate more recent demographic information compared to mitochondrial markers. Novel microsatellite markers were developed in this study. Considering that *K. koreana* is a rare species worldwide that needs careful management, the results of this study provide important baseline data that will inform future conservation strategies.

## Methods

### Sample collection

*Karsenia koreana* individuals were collected from April 2018 to August 2019 throughout all regions where this species is known to exist (Fig. 1). A tip of tail was clipped for each individual and stored in 70% EtOH until DNA extraction. After sample collection, the living individuals were released back to the original site of capture. A total of 204 individuals from 11 populations, whose localities are geographically separated, were used for this study (Table 1).

**Table 1:** Information of *Karsenia koreana* populations and samples analyzed in this study.

Location	Code	Longitude	Latitude	Altitude (m)	Sample size	
					mitochondrial DNA	Microsatellite
DaeJeon	DJ	36.18826	127.3337	277	10	22
GongJu	GJ	36.35361	127.2201	253	9	9
BoEun	BE	36.52549	127.8582	328	8	25
JeCheon	JC	36.86138	128.0549	370	9	20
PyeongChang	PC	37.47148	128.5436	802	9	24
JeongSeon	JS	37.20680	128.7156	691	9	18
SamCheok	SC	37.09339	129.1771	310	6	6
HapCheon	HC	35.80649	128.0963	673	7	20
JinAn	JA	35.76491	127.4747	424	9	25
JeongEup	JE	35.48768	126.8939	272	10	16
GwangYang	GY	35.11225	127.6029	778	9	19
Total					95	204

### Ethics declaration

The sampling protocol was approved by the Korean Ministry of Environment. The sample collection in the field was carried out under the strict guideline on ethical animal experimentation protocols provided by Seoul National University Institutional Animal Care and Use Committee (SNUIACUC) and the guideline provided in the permits conforming to the Wildlife Protection and Management Act of the Korean Ministry of Environment.

### Laboratory protocols for the loci characterization

Genomic DNA was extracted using a DNeasy Blood and Tissue Kit (QIAGEN, Hilden, Germany) following the manufacturer's protocol. The quantity and quality of each DNA sample were assessed using an Epoch Microplate Spectrophotometer (BioTek, Winooski, VT, USA). Successfully extracted DNA samples were diluted to 10–20 ng/μL and stored at − 20 ℃ until use for the genetic analyses.

For the two mitochondrial loci, two novel primers sets were developed (Supplementary Information Table S1) utilizing Primer3^31^ in Geneious Prime 2019.1.3 (https://www.geneious.com) and Primer Premier 6.25 (PREMIER Biosoft, Palo Alto, CA, USA). Primers KkCOI_F6 and KkCOI_R7 were designed to amplify a complete COI gene (1,551 bp; Supplementary Information Table S1), while primers KtRNA_25F and KkCytb_R7 were designed and used to amplify a nearly complete Cyt *b* gene (1,116 bp; Supplementary Information Table S1). Polymerase chain reaction (PCR) for both mitochondrial loci were performed in a volume of 30 μL containing 1X *Ex Taq* buffer with 2 mM MgCl_2_, 0.2 mM dNTP mixture, 1 μL of 10 μM forward and reverse primers each, 1 U *Ex Taq* polymerase (Takara Bio, Shiga, Japan) and 1 μL of template DNA using a TaKaRa PCR Thermal Cycler Dice Gradient (Takara Bio). The thermal cycle profile for the loci consisted of an initial denaturing at 94 ℃ for 5 min; 35 cycles of a denaturing at 94 ℃ for 45 s, an annealing at 60 ℃ for 1 min and an extension at 72 ℃ for 3 min (for Cyt *b*: 65 ℃ for 5 min); and a final extension at 72 ℃ for 10 min. This extension step at lower temperature for longer time of Cyt *b* was necessary, probably due to the putative secondary structure of flanking regions by poly A and T^32^, which was verified in our preliminary experiments. The PCR products were purified and sequenced by Macrogen Inc. (Seoul, South Korea) and Cosmo Genetech Inc. (Seoul, South Korea) on an AB 3730xl DNA analyzer (Applied Biosystems, Foster City, CA, USA).

The novel microsatellite markers were isolated by Macogen. Whole genomic DNA was subjected to pair-end sequencing using a MiSeq platform (Illumina, San Diego, CA, USA). The extracted reads were processed to remove adaptor sequences, and RepeatModeler^33^ (http://www.repeatmasker.org/) was used for the identification of repetitive DNA sequences. SSR Finder^34^ (ftp://ftp.gramene.org/pub/gramene/archives/software/scripts/) was used for annotating reads containing simple repeat sequence motifs. A total of 52 candidate markers (32 tetra-, 10 tri- and 10 dinucleotide repeats) were selected for the testing of amplification and polymorphism with an 8-individual test panel (four populations, two samples each). Fourteen microsatellite markers were chosen to genotype the remaining samples. Microsatellite PCR amplification was performed in 20 μL volume consisting of 1X *Taq* buffer with 2 mM MgCl_2_, 0.2 mM dNTP mixture, 0.5 μL of 10 μM forward and reverse primers each, 1 U *i-StarTaq* polymerase (iNtRON Biotechnology, Seongnam, South Korea) and 1 μL of template DNA using a TaKaRa PCR Thermal Cycler Dice Gradient (Takara Bio). The PCR conditions consisted of an initial denaturing at 94 ℃ for 5 min; 20 cycles of denaturing at 94 ℃ for 20 s, touchdown annealing at 60–50 ℃ for 20 s and an extension at 72 ℃ for 20 s; additional 20 cycles of denaturing at for 20 s, annealing at 50 ℃ for 20 s and an extension at 72 ℃ for 20 s; and a final extension at 72 ℃ for 7 min. Amplified PCR products were genotyped on an ABI 3730xl by NICEM Inc. (Seoul, South Korea). Genotype quality check and peak calling were performed using GeneMapper 3.7 (Themo Fisher Scientific, Waltham, MA, USA).

### Mitochondrial diversity and structure

The quality checking, trimming and editing of the mitochondrial sequence data were carried out using Geneious Prime. The sequences were aligned using ClustalW^35^ under the default setting, implemented in MEGA X^36^. No gaps or ambiguous bases were found.

We estimated the following parameters for each population based on the concatenated sequences of COI and Cyt *b* using DnaSP 5.10.01^37^; number of haplotypes, haplotype diversity (*H*_d_)^38^, nucleotide diversity (π)^38^ and sequence diversity (*k*, average number of nucleotide differences)^39^. Haplotype sequences of the two loci obtained in this study were deposited in GenBank (Accession Nos. MT106778–MT106825; Supplementary Information Table S2). The number and frequency of private haplotypes (_*P*_H) were also calculated based on the haplotype data. The level of population divergence (*Φ*_*ST*_^40^) and the average number of nucleotide differences per site between populations (*D*_*XY*_^38^) were estimated using Arlequin 3.5.2.2^41^ and DnaSP 5.10.01^37^, respectively.

### Phylogenetic analyses

The haplotype network was reconstructed using a median-joining approach in NETWORK 10.0.0.0^42^ (http://www.fluxus-engineering.com). For phylogenetic tree reconstruction, we used *Hydromantes brunus* (GenBank accession No. AY728234.1) as an outgroup species, as it is the most probable sister taxon of *K. koreana*^18,22,43^. Prior to phylogenetic tree reconstruction, the selection of partitioning scheme and substitution models were performed based on a greedy algorithm^44^ with the Bayesian Information Criterion (BIC) using PartitionFinder 2.1.1^45^. Phylogenetic trees were reconstructed based on maximum likelihood (ML) and Bayesian inference (BI) methods were implemented in RaxML^46^ on the CIPRES platform^47^ and MrBayes 3.2.6^48^, respectively.

For the ML analysis, we applied 'ML Thorough Boostrap' workflow with 1000 bootstrap replicates under the GTR + I substitution model^49^. For the BI analysis, two independent Metropolis Coupled Markov Chain Monte Carlo (MCMC) runs of 10^7^ generations were conducted using four chains per run with three heated and one cold (temperature set to 0.1). We sampled trees every 500 generations and discarded 25% of the trees as burn-in. We used TRACER 1.7.1^50^ to assess convergence of parameter estimates and posterior probabilities. The remaining trees were summarized to obtain a 50% majority-rule consensus tree. The tree was visualized using FigTree 1.4.3 (http://tree.bio.ed.ac.uk/software/figtree/).

### Divergence time estimation and historical demography

Divergence times among populations of *K. koreana* were estimated using BEAST 2.6.1^51^ based on the concatenated dataset of COI and Cyt *b*. Five outgroup taxa were used; *Hydromantes brunus* (AY728234.1), *Aneides hardii* (NC_006338.1), *Desmognathus wrighti* (NC_006337.1), *Desmognathus fuscus* (NC_006339.1) and *Ensatina eschscholtzii* (NC_006328.1). For molecular clock calibration, we adopted four molecular dating constraints (*Karsenia-Aneides*: 40.5 Ma, *Aneides-Hydromantes*: 39.4 Ma, *Aneides-Desmognathus*: 37.8 Ma, *Ensatina-Hydromantes*: 37.9 Ma) provided in Shen et al.^19^. The means and standard deviations of the normal distribution for these priors were chosen to reflect arithmetical medians of 95% credible intervals.

Given the population-level phylogenetic relationships among *K. koreana* lineages may be relatively close, we assigned a strict clock model^52^, coalescent constant population model and GTR + I + G substitution model under the default setting. Nested sampling algorithm^53,54^ posteriorly confirmed this combination of model parameters among multiple alternatives. The MCMC of BEAST consisted of three independent runs of 10^8^ generations with sampling log and tree files every 1000 generations. After running, the convergence of chains was verified and burn-in periods were determined in TRACER to ensure the effective sample sizes (ESS) for all parameters were over 200. Three independent MCMC runs were combined in LogCombiner 2.6.1. Predetermined 10% burn-in trees were discarded and the final phylogenetic tree was annotated by maximum clade credibility type and median node heights in TreeAnnotator 2.6.0. The tree was drawn by FigTree 1.4.3.

Extended Bayesian skyline plot (EBSP)^55^ was implemented in BEAST to estimate historical demographic change. An unpartitioned HKY + I substitution model^56^ was applied, and prior selection, clock model, parameter settings and MCMC setup were the same as those used for the divergence time estimation. To incorporate a time scale into the analysis, we assigned two additional priors; normal distributed clock rate (mean: 6.8629E-3, sigma: 5.9743E-4) and lognormal distributed MRCA prior (mean: 2.318, sigma: 0.3031). The result was plotted with log-scale population size by time (Ma) using R package^57^ based on 'plotEBSP' function provided in BEAST. The likelihood of historical demographic expansion was tested based on Tajima's *D*^58^ and Fu's *F*_S_^59^, which was implemented in DnaSP 5.10.01^37^ with 10,000 replicates.

### Microsatellite diversity

Micro-Checker 2.2.3^60^ was used to detect null alleles, large allelic dropout and potential scoring errors. Hardy–Weinberg equilibrium and the likelihood of linkage disequilibrium among selected marker loci were examined using Fisher's exact test under 10,000 dememorization, 100 batches and 5000 iterations per batch, as implemented in Genepop 4.7.2^61^. We calculated the following parameters using GenAlEx 6.503^62^: number of alleles (*N*), number of effective alleles (*N*_a_), observed heterozygosity (*H*_O_), expected heterozygosity (*H*_E_), and fixation index (*F*_IS_) for each locus and population and pairwise relatedness among samples to avoid duplicates. The signature of historical bottleneck was tested utilizing 1000 iterations of mode-shift at IAM, SMM and TPM (70% SMM, 30% variance) using BOTTLENECK 1.2.02^63^. Garza-Williamson index (*M*-ratio)^64^ was quantified using AGARst 3.3^65^. When the *M*-ratio dropped significantly below 0.68 according to the traditional method, the population was considered to have experienced a severe historical population decline.

### Microsatellite population structure

Pairwise *F*_*ST*_^66^ and -*R*_*ST*_^67^ values were computed using FSTAT 2.9.4^68^ and Arlequin 3.5.2.2^41^ with 1000 permutations. Isolation by distance (IBD), the relationship between geographic distance and Slatkin's linearized *F*_*ST*_ (*F*_*ST*_ / (1 − *F*_*ST*_))^69^, was analyzed and visualized using GenAlEx 6.503^62^. Covariance-standardized Principal Coordinate Analysis (PCoA) was performed to identify the distribution of genetic characteristics among populations at the individual level using GenAlEx 6.503^62^. Genetic barriers against gene flow among populations were mapped using BARRIER 2.2^70^ according to Monmonier's maximum difference algorithm^71^ with 1000 bootstrapped Nei's chord distance (*D*_*A*_)^72^ and *F*_*ST*_ matrices generated by MSA 4.05^73^ and R package FinePop^74^, respectively. The level and pattern of population structure was visualized using Structure 2.3.4^75^ that was implemented with 10 iterations of 10^5^ MCMC generations (including 10,000 burn-in) from *K* = 1 to *K* = 11 (the number of populations). The optimal *K* was determined on STRUCTURE HARVESTER web 0.6.94^76^ based on Evanno method^77^.

To infer the evolutionary history of *K. koreana* populations, over 100 scenarios were predefined to include both northward and southward dispersal patterns of different geographically probable orders among genetic clusters. The scenarios were examined in DIYABC 2.1.0^78^ in a tournament fashion. Genetically close populations were grouped into a single cluster to lower the computational load, resulting in seven populations/clusters. More than 10^5^ simulations per scenario were performed and scenarios were compared based on posterior probabilities. If the comparison results of the direct and logistic approaches were inconsistent, the confidence of high-ranked scenarios were evaluated again to discriminate among them. For the last two selected scenarios, 10^6^ simulations were implemented for each to estimate the parameters of effective population size and branching point.

## Results

### Mitochondrial analysis

A total of 38 haplotypes were identified from the 11 K*. koreana* populations, 37 of which were unique to an individual population. The *H*_d_ value of the species in total was identified (0.942), while its π value was relatively low (0.00995). The ratios of private haplotypes (*N*_*P*_ / *N* and _*P*_*H* / *H*) were relatively low in populations [BE], [JS], [PC] and [SC] (Supplementary Information Table S3). Population [GY] was the most genetically distinct of all the populations, followed by population [HC] (Supplementary Information Table S4). The levels of divergence among populations [JS], [PC] and [SC] were negligible (Supplementary Information Table S4).

**Figure 2:**
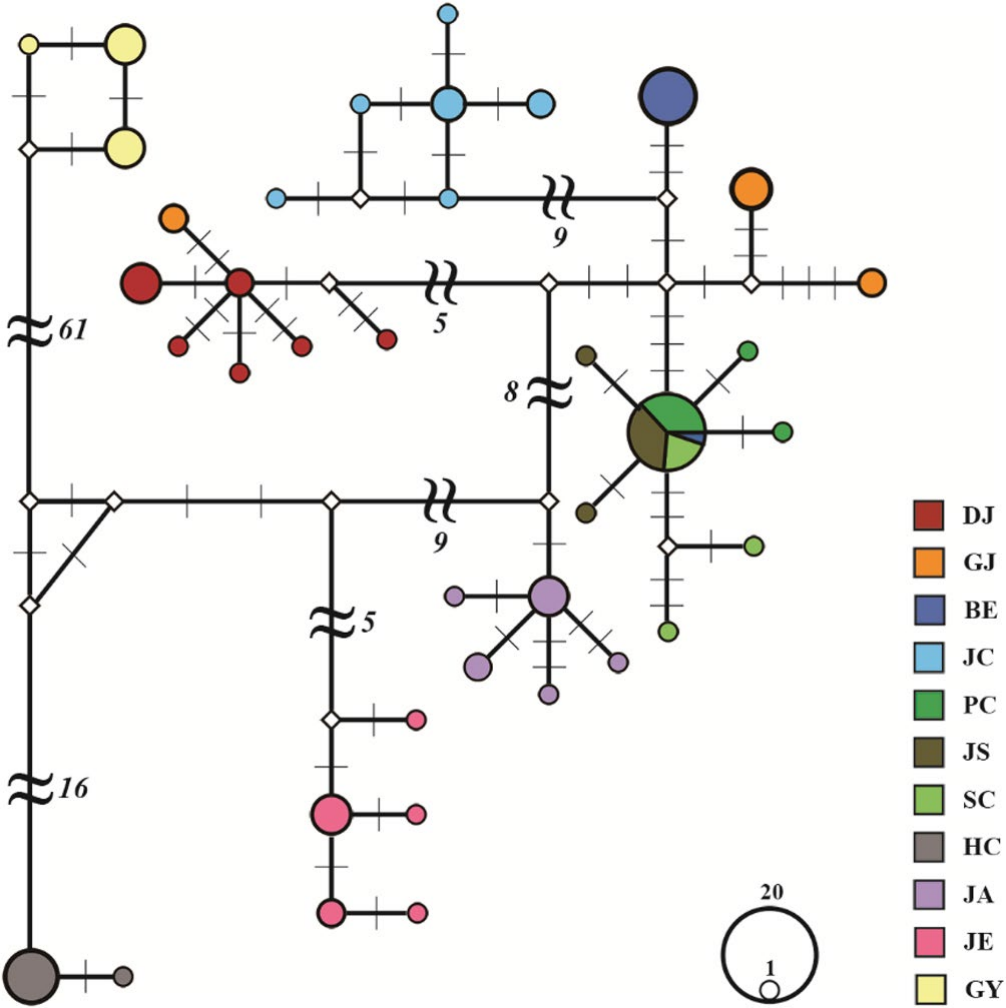
Haplotype network among *Karsenia koreana* populations estimated using concatenated sequences of cytochrome *c* oxidase I and cytochrome *b*. Different colors are assigned to each population, and the size of a circle is proportional to the haplotype frequency. Mutation steps are indicated by vertical lines, or numbers in case of more than five. Population codes follow Table 1.

Although populations [PC], [JS] and [SC] shared haplotypes, the other populations had their own unique haplotypes. The haplotypes of the populations in close geographical proximity tended to be close to each other in the haplotype network (Fig. 2).

The overall topology of ML and BI trees were nearly identical (Supplementary Information Figure S1 and S2). Consistent with the haplotype network results, the ML and BI trees grouped *K. koreana* populations into nine clusters, [BE + PC + JS + SC], [GJ], [BE], [JC], [GJ + DJ], [JA], [JE], [HC] and [GY]. Cluster membership tended to be related to geographical locations, with some exceptions. In particular, population [GY] was consistently distinct and separated from the other populations, followed by [HC] and [JE], the second and third most distinct populations.

**Figure 3:**
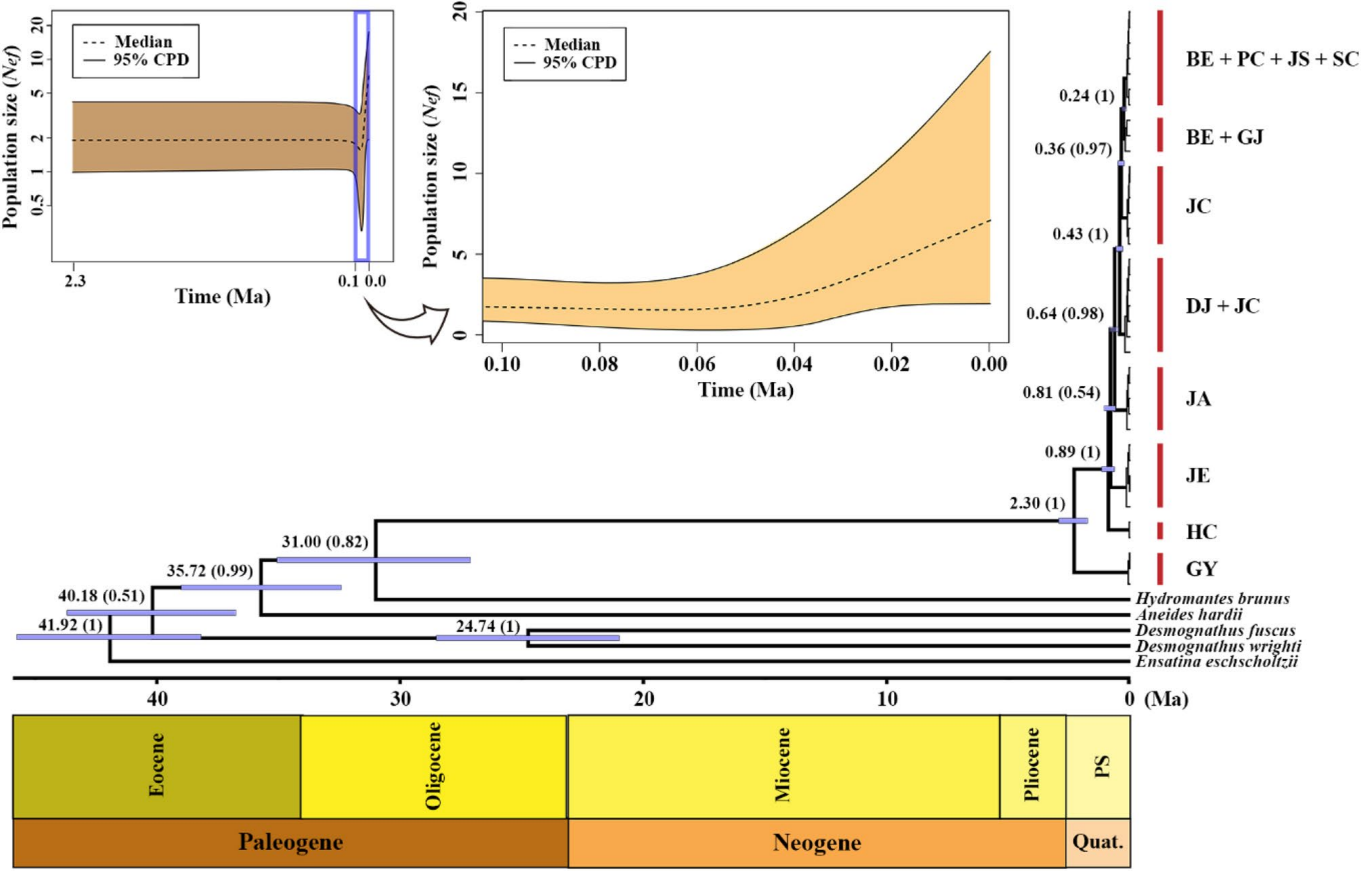
Time-calibrated Bayesian tree reconstructed using BEAST based on the concatenated sequences of cytochrome *c* oxidase I and cytochrome *b*, given with a geological time scale chart under the time scale bar (abbreviations: PS: Pleistocene, Quat.: Quaternary). At each major node, median divergence date is represented in Ma (million years ago) and the posterior probability of the date is indicated in brackets. The insets show Bayesian skyline plots of 0–2.3 Ma range (top left) and the 0–0.1 Ma range is magnified (top right). X and Y axes represent time (Ma) and log-scale effective population size (*Nfe*), respectively. Population codes follow Table 1.

Recovered divergence dates of prior calibration constraints were within the 95% credible intervals of dates recovered in a previous study (*Karsenia*-*Aneides*: 35.39 Ma, *Aneides*-*Hydromantes*: 36.18 Ma, *Aneides*-*Desmognathus*: 40.71 Ma, *Ensatina*-*Hydromantes*: 41.08 Ma)^19^. In our results, the first split between population [GY] and the others was estimated to have occurred around 2.30 Ma (95% highest posterior density range: 1.74 ~ 2.92 Ma) (Fig. 3). At approximately 1 Ma after this divergence, [HC] and [JE] split off, followed chronologically by [JA], [GJ + DJ], [JC], [BE] and [PC + JS + SC].

The historical demography analysis inferred a continuously stable population size, represented by a typical J-shaped skyline plot that reflects faster molecular evolution on a shorter time scale^79^ (Fig. 3). Tajima's *D* and Fu's *F*_S_ tests were conducted for each mitochondrial locus and the results are summarized in Supplementary Information Table S3. None of these tests rejected the null hypothesis of neutral evolution with constant population size.

### Microsatellite analysis

Ten tetra-, two tri- and two di-nucleotide microsatellite markers were chosen, as they successfully amplified and contained adequate levels of polymorphism within and among populations. Although no large allelic dropout was detected, the presence of null alleles was suspected in all loci analyzed. Null alleles were found in only one or two populations across the loci and were not found associated with any specific population. This suggests that the potential for substructure or inbreeding within populations was low. The overall null allele frequencies in loci K1039 and K1040 were higher than that in the low-frequency zone (see Dakin and Avise^80^), and loci K1011 and K1040 had null alleles in two or more populations. We included these three loci (K1011, K1039 and K1040) in subsequent analyses as the analyses results did not differ with or without these loci (see Oromi et al.^81^). We did not detect any signature of linkage disequilibrium among the loci used (data now shown).

Overall, the 14 microsatellite loci exhibited moderately high genetic diversity (Supplementary Information Table S5). The tetra-nucleotide loci generated higher levels of diversity (*H*_E_ = 0.841 − 0.921), whereas the di- and tri-nucleotide loci showed slightly lower levels of diversity (*H*_E_ = 0.677 − 0.874) and were associated with high *F*_IS_ values, indicating the likely presence of null alleles (Supplementary Information Table S5). The levels of genetic diversity among the populations differed, especially among populations [PC], [JS] and [SC] (Table 2). No signature of genetic bottleneck was detected in any of the populations (Supplementary Information Table S6). Moreover, all populations had an *M*-ratio greater than 0.68, considered as the threshold level of historical bottleneck (Supplementary Information Table S6).

**Table 2:** Summary statistics of 11 *Karsenia koreana* populations throughout South Korea based on microsatellite data. Population codes follow Table 1. The value for each statistic is followed by the standard error in parentheses.

Population	*N*	*N*_a_	*N*_e_	*H*_O_	*H*_E_	u*H*_E_	*F*_*IS*_
DJ	22	9.857 (1.199)	6.119 (0.758)	0.727 (0.059)	0.769 (0.054)	0.787 (0.055)	0.050 (0.037)
GJ	9	7.286 (0.759)	5.358 (0.627)	0.730 (0.060)	0.758 (0.043)	0.803 (0.045)	0.043 (0.057)
BE	25	8.643 (0.708)	5.159 (0.619)	0.680 (0.059)	0.728 (0.055)	0.743 (0.056)	0.078 (0.034)
JC	20	7.571 (1.026)	4.318 (0.691)	0.671 (0.048)	0.683 (0.052)	0.700 (0.054)	0.002 (0.031)
PC	24	3.929 (0.559)	2.462 (0.372)	0.449 (0.082)	0.457 (0.079)	0.467 (0.080)	0.041 (0.048)
JS	18	5.357 (0.617)	2.720 (0.407)	0.480 (0.066)	0.510 (0.068)	0.525 (0.070)	0.038 (0.038)
SC	6	3.929 (0.385)	2.732 (0.332)	0.476 (0.067)	0.551 (0.060)	0.601 (0.065)	0.124 (0.085)
HC	20	8.643 (0.905)	5.002 (0.616)	0.654 (0.058)	0.723 (0.055)	0.742 (0.057)	0.086 (0.041)
JA	25	10.071 (1.112)	5.639 (0.677)	0.697 (0.069)	0.747 (0.059)	0.762 (0.061)	0.059 (0.054)
JE	16	7.143 (1.079)	4.345 (0.741)	0.647 (0.082)	0.629 (0.076)	0.650 (0.078)	− 0.040 (0.048)
GY	19	8.000 (0.646)	5.120 (0.518)	0.744 (0.045)	0.768 (0.033)	0.788 (0.034)	0.029 (0.040)

Abbreviations: Sample size (*N*), number of alleles (*N*_a_), effective number of alleles (*N*_e_), observed heterozygosity (*H*_O_), expected heterozygosity (*H*_E_), unbiased expected heterozygosity (u*H*_E_) and fixation index (*F*_*IS*_).

**Figure 4:**
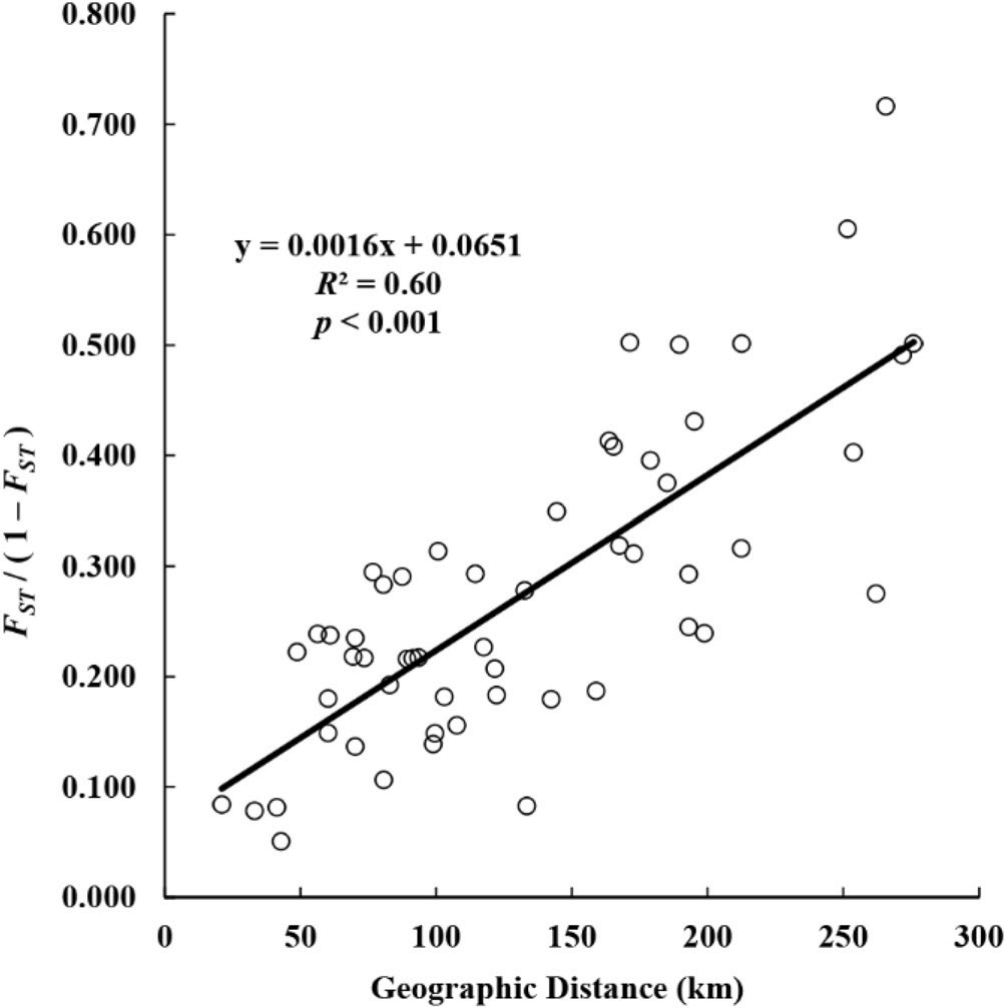
Isolation by distance plot among *Karsenia koreana* populations estimated based on microsatellite genotyping results. X and Y axis indicate geographic distance (km) and Slatkin's linearized F_*ST*_ (F_*ST*_ / ( 1 − F_*ST*_ )), respectively.

Based on pairwise-*F*_*ST*_ and -*R*_*ST*_, the overall level of genetic differentiation among populations was fairly high (Table 3). Since the overall values of pairwise-*R*_*ST*_ were much larger than those of pairwise-*F*_*ST*_ (Table 3), this genetic differentiation is likely due to spatial isolation between populations rather than genetic drift, resulting from population size fluctuations. In particular, populations [JA], [JE] and [GY] exhibited higher levels of genetic differentiation compared to those of the other populations (Table 3). Genetic differentiation among populations was correlated with geographic distance. For example, in each set of populations comprising [DJ], [GJ], [BE] and populations [PC], [JS], [SC], the populations are in close spatial proximity, and they show exceptionally low levels of genetic differentiations between them (Table 3). This pattern was also evident in the IBD test results (Fig. 4).

**Table 3:** Pairwise genetic differentiation among 11 *Karsenia koreana* populations estimated from microsatellite data. Population codes follow Table 1. Estimates of *R*_*ST*_ and *F*_*ST*_ appear above and below the diagonal, respectively.

	DJ	GJ	BE	JC	PC	JS	SC	HC	JA	JE	GY
DJ		0.160	0.261	0.086	0.544	0.501	0.414	0.123	0.733	0.825	0.436
GJ	0.077**		0.389	0.226	0.701	0.640	0.532	0.193	0.692	0.841	0.491
BE	0.130	0.153		0.120	0.343	0.310	0.202	0.259	0.715	0.828	0.535
JC	0.122**	0.179	0.075		0.444	0.408	0.317	0.164	0.735	0.830	0.502
PC	0.283	0.334	0.172	0.221		0.000^NS^	0.150	0.486	0.761	0.904	0.768
JS	0.242	0.292	0.135	0.190	0.073		0.060*	0.440	0.721	0.877	0.728
SC	0.197	0.227*	0.076**	0.154	0.120	0.048^NS^		0.334	0.651	0.851	0.661
HC	0.096	0.129	0.161	0.185	0.334	0.290	0.238**		0.664	0.743	0.276
JA	0.182	0.179	0.178	0.218	0.334	0.301	0.240	0.193		0.399	0.682
JE	0.225	0.239	0.259	0.273	0.417	0.377	0.329	0.227	0.192		0.818
GY	0.155	0.152	0.158	0.193	0.334	0.287	0.216	0.178	0.178	0.228	

All estimated values significantly deviated from zero (*p* < 0.001) except those denoted by **(*p* < 0.01), *(*p* < 0.05) and 'NS'.

**Figure 5:**
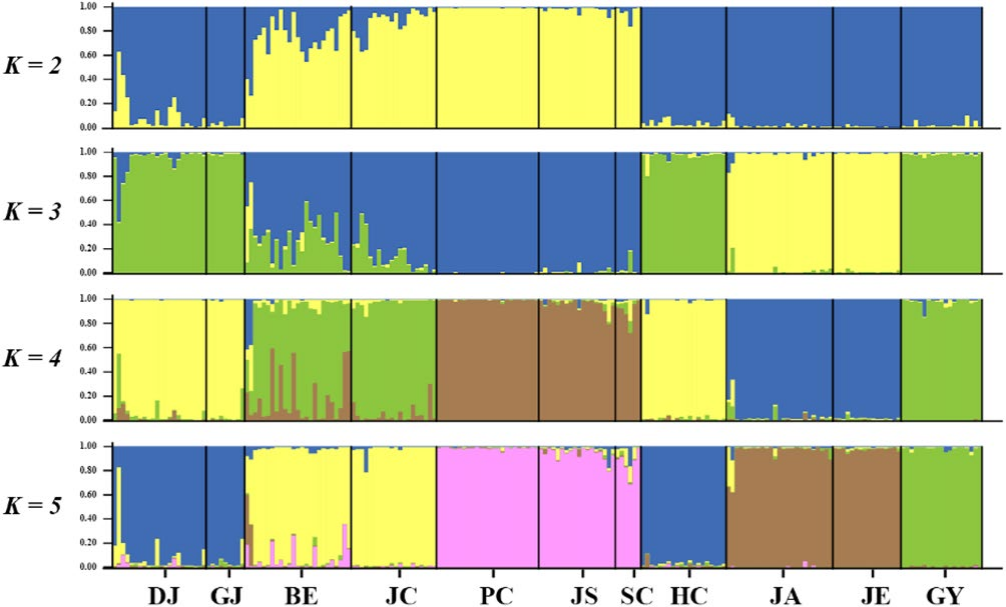
Bayesian genetic structure among *Karsenia koreana* populations estimated based on the microsatellite data using Structure. Since the program could not identify one optimal number of genetic clusters, four serial plots from *K* = 2 to 5 are presented. Population codes follow Table 1.

PCoA grouped populations into four clusters: [JS + PC + SC], [BE + JC], [DJ + GJ + GY + HC] and [JA + JE], with slight overlaps between groups (Supplementary Information Figure S3). Group [JA + JE] was clearly separated from the other groups without overlap (Supplementary Information Figure S3). The membership of each of these four groups is related to spatial proximity (Supplementary Information Figure S3). In our Bayesian Structure analysis, the delta *K* method implemented in Structure Harvester was unable to identify an optimal number of genetically distinguishable clusters. The result, *K* = 2, is the minimal value, and may be a result of underestimation^77,82^. The overall clustering pattern generated by PCoA is reflected in the Structure analysis (Fig. 5), except that populations [JA] and [JE] are distinct from each other (Fig. 5). Bootstrapped values of *D*_*A*_ provide estimates of barriers to gene flow between populations; based on this value, populations [JA] and [JE] are completely isolated from the other populations (Supplementary Information Figure S4). Conversely, no barrier to gene flow was detected among populations [BE], [JC], [PC], [JS] and [SC] (Supplementary Information Figure S4).

DIYABC found two scenarios having the highest statistical probability (Supplementary Information Table S7 and Figure S5). In the first scenario (Supplementary Information Table S7 and Figure S5), [PC + JS + SC], [JA] and [GY] diverged from a common ancestor approximately 5,380 generations ago, while [JE] diverged approximately 2,950 generations ago from [JA]. The remaining populations diverged into [PC + JS + SC], [DJ + GJ], [BE + JC] and [HC] approximately 2,360 generations ago. In the second scenario (Supplementary Information Table S7 and Figure S5), [JA] and [GY] diverged from a common ancestor approximately 13,200 generations ago and [JE] branched off from [JA] approximately 2,090 generations ago. The remaining populations diverged into [JS + PC + SC], [DJ + GJ], [BE + JC] and [HC] approximately 5,710 generations ago. [JS + PC + SC] and [BE + JC] diverged recently, approximately 263 generations ago. Logistic regression and confidence testing (linear discriminant analysis) results indicate that the second scenario is more probable (Supplementary Information Table S8 and Figure S5).

## Discussion

In general, *Karsenia koreana* showed distinct population genetic structure. The various approaches that we implemented consistently show clear genetic clustering. Both mitochondrial and microsatellite data generally demonstrate a positive correlation between pairwise genetic differentiations and pairwise geographic distances among populations in general. Moreover, each of the southern populations ([GY], [HC], [JE] and [JA]) was differentiated from the other populations. Furthermore, both the BEAST divergence and DIYABC analyses indicate that the most likely evolutionary scenario consists of northward divergence events originating from a southernmost population [GY].

### Population genetic diversity and structure

The range of values of mitochondrial diversity of *K. koreana* (π = 0.00011–0.00237) are relatively lower than those of other plethodontid species, e.g., European *Hydromantes* species (π = 0.003–0.037)^83,84^ and *Gyrinophilus porphyriticus* (π = 0.031)^85^; and East Asian salamander species, e.g., *Pachyhynobius shangchengensis* (π = 0.0345)^86^ and *Hynobius quelpaertensis* (π = 0.0174–0.0214)^87^. However, the frequency of heterozygotes in the microsatellite data (*H*_*O*_ = 0.449–0.744) suggests that *K. koreana* is more genetically diverse than other plethodontid species, e.g., *Plethodon cinereus* (*H*_*O*_ = 0.189–0.420)^88^; and other Northeast Asian salamanders, e.g., Japanese *Onychodactylus* species (*H*_*O*_ = 0.28–0.61)^89^. Even at the population level, mitochondrial and microsatellite data show different patterns of genetic diversity, although differences in sample sizes should be taken into account. For example, populations located in the Taebaek Mountain range ([PC], [JS] and [SC]) showed the lowest levels of microsatellite diversity, while the mitochondrial diversity of these populations was slightly higher than those of populations [BE] and [HC], which had shallow levels of genetic differentiation.

Overall, the results of genetic structure at the population-level were congruent for mitochondrial DNA and microsatellite data. The spatial location of the populations correlated well with the genetic distance between populations using both genetic markers. Considering that this species does not actively move long distances and tends to stay in a limited habitat, it is unlikely that gene flow over long distances occurs. However, results from both mitochondrial and microsatellite loci, suggest strong gene flow has occurred between populations [PC], [JS] and [SC]. This is probably because populations occupying a complex, continuous mountain terrain have access to a shady, humid environment that allows for the relatively active movement of individuals.

An incongruence between the population structures results based on the two genetic markers (microsatellite, mitochondrial DNA) should be noted. Microsatellite analysis via Structure and PCoA clustered together populations [DJ + GJ] and [HC], while [HC] was relatively well separated from the other populations based on mitochondrial analyses (the haplotype network and phylogenetic trees). In the mitochondrial results, [GJ] was relatively well separated from [DJ]; yet one haplotype of [GJ] grouped with the [DJ] haplotypes. However, these two genetic markers exhibit different modes of inheritance and act on different evolutionary time scales, thus it is not uncommon to observe such incongruences, and many theories and hypotheses provide plausible explanations for such findings^90–93^.

Microsatellites and mitochondrial DNA provided different estimates in the effective population size, a finding that should be noted considering that this species is endangered^90,94^. Fluctuation by genetic drift is likely to occur in mitochondrial data, which provided smaller estimates of effective population size. Thus genetic drift may have caused the differences in the patterns of diversity and structure between the two markers. Historical demographic analyses based on both of the markers used in this study was unable to reject the null hypothesis (a constant population size) and additionally indicated small population sizes.

### Phylogeography

During the glacial advances of the Pliocene and the glacial cycles of the Pleistocene, animal species in the Northern Hemisphere were restricted to a southern refugium^3,95^. Later, the retreating ice sheets allowed many animals to recolonize northward. However, if the environments in the southern refugium contain sufficient resources, populations may stay in the refugium and/or adapt to the surrounding areas, rather than recolonize their original habitats. Gómez and Lunt^4^ proposed that within a big refugium composed of complex mountain ranges, multiple small refugia could form. The idea of small refugia existing within a big refugium has been proposed in many studies^5,96–98^, including empirical studies on plethodontids^99,100^ and other East Asian animals^101–105^. This scenario also provides a reasonable interpretation of our data, i.e., genetically distinct *K. koreana* populations exist in small refugia that are distributed in or around the mountainous terrain of the Korean Peninsula, which in total may be considered one big refugium.

Previous studies inferred that the ancestor of *K. koreana* crossed from western North America to East Asia approximately 65 Ma^19,22^. Since *K. koreana* is confined to the Korean Peninsula, we hypothesize that the current populations are the surviving relics of the species, and the present-day population genetic structure is the result of a relatively recent northward expansion from a southernmost population. With a few exceptions, the results of Structure, PCoA and haplotype network analyses indicate that the population structure of this species was formed in a unidirectional fashion that matches the terrain of the Korean Peninsula's mountain range. The BEAST divergence tree and the most probable DIYABC evolutionary scenario from mitochondrial and microsatellite data, respectively, are both consistent in their support for a historical northward dispersal and divergence from the southernmost population [GY]. Based on mitochondrial data, most *K. koreana* populations diverged approximately after 1 Ma; and the pattern of isolation by distance from genetically more differentiated southern populations [GY], [HC], [JE] and [JA] further support this idea. Taken overall, these data suggest that *K. koreana* populations that survived in the southern part of the peninsula throughout the Pleistocene glaciation migrated northward and most recently recolonized favorable habitats along the complex mountain ranges. To evaluate this hypothesis, future work should be done to locate additional populations in the northern part of the Korean Peninsula (i.e., North Korea), and if there are, determine if they exhibit the same evolutionary pattern. Additionally, one outlier that needs further investigation is the divergence between [GY] and all the other populations, estimated to have occurred approximately 2.3 Ma (see also Fig. 3). To clarify this, additional phylogenetic analyses are needed using different types of genetic markers, such as nuclear genes or ddRAD markers.

### Implications for conservation

The delimitation of a species′ 'Management Unit' (MU) is important in conserving its current population genetic diversity^106^. A MU is defined as "a functionally independent population of a species that has formed under restricted levels of gene flow."^106^. Our results indicate that there are seven *K. koreana* MUs: [GY], [HC], [JE], [JA], [DJ + GJ], [BE + JC] and [PC + JS + SC]. Such a proposal has an important implication for the conservation of this species.

Presently, the IUCN red list designates *Karsenia koreana* as a species of "Least Concern,"^23^ mostly because it is widespread on the Korean Peninsula. However, our data suggest that historical gene flow between the populations was low, isolating populations and increasing the influence of genetic drift. Therefore, although this species is widespread, the average effective population size is very small. Conserving this species requires special conservation strategies that take into account the preservation of all seven MUs.

## Supplementary Information

Supplementary Information

## Data Availability

All data needed to evaluate the conclusions in the paper are present in the paper and the Supplementary Information materials. Sequences in the paper have been deposited at GenBank (Accession Nos. MT106778–MT106825).
