# Supplementary material for: The Asian plethodontid salamander preserves historical genetic imprints of recent northern expansion
Source: Sci Rep. 2021 Apr 28;11:9193. doi: 10.1038/s41598-021-88238-z (PMC8080585; doi:10.1038/s41598-021-88238-z)
Supplement: Supplementary file 1 — Supplementary Information [file 41598_2021_88238_MOESM1_ESM.docx]

**Supplementary Information for:**

**The Asian plethodontid salamander preserves**

**historical genetic imprints of recent northern expansion**

Jong Yoon Jeon^1^, Ji-hwa Jung^2^, Ho Young Suk^3^, Hang Lee^1^ and Mi-Sook Min^*,1^

**Table of Contents**

| **Table S1** | **Information of primers developed in this study for each mitochondrial DNA locus, cytochrome *c* oxidase I (COI) and cytochrome *b* (Cyt *b*)** | Page 1 |
| --- | --- | --- |
| **Table S2** | **Information of concatenated haplotype composition and GenBank accession numbers for each cytochrome *c* oxidase I and cytochrome *b* haplotype** | Page 2–3 |
| **Table S3** | **Genetic variability and historical demography factors of *Karsenia koreana* populations calculated based on concatenated sequences of cytochrome *c* oxidase I and cytochrome *b*** | Page 4 |
| **Table S4** | **Pairwise genetic differentiation among eleven *Karsenia koreana* populations estimated using concatenated sequences of cytochrome *c* oxidase I and cytochrome *b*** | Page 5 |
| **Table S5** | **Information and summary statistics of the 14 microsatellite markers used in this study** | Page 6 |
| **Table S6** | **Results of historical demography analyses of *Karsenia koreana* based on 14 microsatellite loci** | Page 7 |
| **Table S7** | **Parameter estimation of two chosen DIYABC scenarios among seven *Karsenia koreana* clusters based on microsatellite data** | Page 8–9 |
| **Table S8** | **Confidence evaluation of two chosen DIYABC scenarios among seven *Karsenia koreana* clusters based on microsatellite data** | Page 10 |
| **Figure S1** | **Maximum likelihood (ML) tree of *Karsenia koreana* populations reconstructed based on the concatenated sequences of cytochrome *c* oxidase I and cytochrome *b* using RaxML** | Page 11 |
| **Figure S2** | **Bayesian inference (BI) tree of *Karsenia koreana* populations reconstructed based on the concatenated sequences of cytochrome *c* oxidase I and cytochrome *b* using MrBayes** | Page 12 |
| **Figure S3** | **Covariance-standardized PCoA plot showing genetic differentiation based on microsatellite *F_ST_* values among *Karsenia koreana* populations** | Page 13 |
| **Figure S4** | **The results of the BARRIER analysis for *Karsenia koreana* populations estimated based on (a) bootstrapped *D_A_*, and (b) bootstrapped *F_ST_*** | Page 14 |
| **Figure S5** | **The products of Approximate Bayesian Computation (ABC) analysis implemented in DIYABC to identify the most likely pattern of historical divergence among populations** | Page 15 |

Table S1. Information of primers developed in this study for each mitochondrial DNA locus, cytochrome *c* oxidase I (COI) and cytochrome *b* (Cyt *b*). Primers are specific for *Karsenia koreana.*

| Name | Primer direction | Sequence | Target gene |
| --- | --- | --- | --- |
| KkCO1_F6 | Forward | CTT GAC CTT TAT TTC CGG GGC | COI |
| KkCO1_R7 | Reverse | TAA GGG GGT GAC AGA TTG GC | COI |
| KtRNA_25F | Forward | AAC CTT TGA CCT GAA AAA TCA GTG TT | Cyt *b* |
| KkCytb_R7 | Reverse | AAA CCA ATG TTT TTC TAA ACT ACA GTG | Cyt *b* |

Table S2. Information of concatenated haplotype composition and GenBank accession numbers for each cytochrome *c* oxidase I and cytochrome *b* haplotype*.*

| Concatenated  haplotype | COI  haplotype | GenBank  Accession No. | Cyt *b*  haplotype | GenBank  Accession No. |
| --- | --- | --- | --- | --- |
| Hap 1 | COI Hap 6 | MT106806 | Cyt *b* Hap 4 | MT106781 |
| Hap 2 | COI Hap 6 |  | Cyt *b* Hap 1 | MT106778 |
| Hap 3 | COI Hap 7 | MT106807 | Cyt *b* Hap 1 |  |
| Hap 4 | COI Hap 6 |  | Cyt *b* Hap 5 | MT106782 |
| Hap 5 | COI Hap 6 |  | Cyt *b* Hap 6 | MT106783 |
| Hap 6 | COI Hap 8 | MT106808 | Cyt *b* Hap 7 | MT106784 |
| Hap 7 | COI Hap 1 | MT106801 | Cyt *b* Hap 1 |  |
| Hap 8 | COI Hap 2 | MT106802 | Cyt *b* Hap 2 | MT106779 |
| Hap 9 | COI Hap 3 | MT106803 | Cyt *b* Hap 3 | MT106780 |
| Hap 10 | COI Hap 4 | MT106804 | Cyt *b* Hap 9 | MT106786 |
| Hap 11 | COI Hap 5 | MT106805 | Cyt *b* Hap 2 |  |
| Hap 12 | COI Hap 16 | MT106816 | Cyt *b* Hap 13 | MT106790 |
| Hap 13 | COI Hap 16 |  | Cyt *b* Hap 14 | MT106791 |
| Hap 14 | COI Hap 16 |  | Cyt *b* Hap 15 | MT106792 |
| Hap 15 | COI Hap 16 |  | Cyt *b* Hap 16 | MT106793 |
| Hap 16 | COI Hap 17 | MT106817 | Cyt *b* Hap 16 |  |
| Hap 17 | COI Hap 18 | MT106818 | Cyt *b* Hap 14 |  |
| Hap 18 | COI Hap 20 | MT106820 | Cyt *b* Hap 2 |  |
| Hap 19 | COI Hap 5 |  | Cyt *b* Hap 20 | MT106797 |
| Hap 20 | COI Hap 21 | MT106821 | Cyt *b* Hap 2 |  |
| Hap 21 | COI Hap 22 | MT106822 | Cyt *b* Hap 2 |  |
| Hap 22 | COI Hap 24 | MT106824 | Cyt *b* Hap 2 |  |
| Hap 23 | COI Hap 25 | MT106825 | Cyt *b* Hap 2 |  |
| Hap 24 | COI Hap 19 | MT106819 | Cyt *b* Hap 17 | MT106794 |
| Hap 25 | COI Hap 19 |  | Cyt *b* Hap 18 | MT106795 |
| Hap 26 | COI Hap 14 | MT106814 | Cyt *b* Hap 10 | MT106787 |
| Hap 27 | COI Hap 4 |  | Cyt *b* Hap 11 | MT106788 |
| Hap 28 | COI Hap 4 |  | Cyt *b* Hap 10 |  |
| Hap 29 | COI Hap 15 | MT106815 | Cyt *b* Hap 10 |  |
| Hap 30 | COI Hap 4 |  | Cyt *b* Hap 12 | MT106789 |
| Hap 31 | COI Hap 9 | MT106809 | Cyt *b* Hap 8 | MT106785 |
| Hap 32 | COI Hap 10 | MT106810 | Cyt *b* Hap 8 |  |
| Hap 33 | COI Hap 11 | MT106811 | Cyt *b* Hap 8 |  |
| Hap 34 | COI Hap 12 | MT106812 | Cyt *b* Hap 8 |  |
| Hap 35 | COI Hap 13 | MT106813 | Cyt *b* Hap 8 |  |
| Hap 36 | COI Hap 23 | MT106823 | Cyt *b* Hap 21 | MT106798 |
| Hap 37 | COI Hap 23 |  | Cyt *b* Hap 22 | MT106799 |
| Hap 38 | COI Hap 23 |  | Cyt *b* Hap 23 | MT106800 |
|  |  |  | Cyt *b* Hap 19^†^ | MT106796 |

^†^ A haplotype not used in the study due to COI amplification failure

Table S3. Genetic variability and historical demographic factors of *Karsenia koreana* populations calculated based on concatenated sequences of cytochrome *c* oxidase I and cytochrome *b.* Population codes follow Table 1.

| Location |  | *N* | *N_p_* | *H* | *_p_H* | *N_p_* / *N* | *_p_H* / *H* | *H*_d_ | π | *k* | Tajima's *D* | Fu's *F_s_* |
| --- | --- | --- | --- | --- | --- | --- | --- | --- | --- | --- | --- | --- |
| DJ |  | 10 | 10 | 6 | 6 | 100% | 100% | 0.844 | 0.00080 | 2.133 |  |  |
| GJ |  | 9 | 9 | 3 | 3 | 100% | 100% | 0.667 | 0.00237 | 6.333 |  |  |
| BE |  | 8 | 7 | 2 | 1 | 88% | 50% | 0.250 | 0.00056 | 1.500 |  |  |
| JC |  | 9 | 9 | 6 | 6 | 100% | 100% | 0.889 | 0.00060 | 1.611 |  |  |
| PC |  | 9 | 2 | 3 | 2 | 22% | 67% | 0.417 | 0.00017 | 0.444 |  |  |
| JS |  | 9 | 2 | 3 | 2 | 22% | 67% | 0.417 | 0.00017 | 0.444 |  |  |
| SC |  | 6 | 2 | 3 | 2 | 33% | 67% | 0.600 | 0.00100 | 2.067 |  |  |
| HC |  | 7 | 7 | 2 | 2 | 100% | 100% | 0.286 | 0.00011 | 0.286 |  |  |
| JA |  | 9 | 9 | 5 | 5 | 100% | 100% | 0.806 | 0.00048 | 1.278 |  |  |
| JE |  | 10 | 10 | 5 | 5 | 100% | 100% | 0.756 | 0.00053 | 1.422 |  |  |
| GY |  | 9 | 9 | 3 | 3 | 100% | 100% | 0.667 | 0.00029 | 0.778 |  |  |
| Total |  | 95 | 76 | 38 | 37 | 80% | 97% | 0.942 | 0.00995 | 26.545 | -0.062  (*p* > 0.10) | -1.034  (*p* > 0.10) |

Abbreviations: total number of individuals (*N*), number of *_p_H* individuals (*N_p_*), number of haplotypes (*H*), number of private haplotypes (*_p_H*), haplotype diversity (*H*_d_), nucleotide diversity (π) and sequence diversity (average number of nucleotide differences; *k*)

**Table S4.** Pairwise genetic differentiation among eleven *Karsenia koreana* populations estimated using concatenated sequences of cytochrome *c* oxidase I and cytochrome *b*. Population codes follow Table 1. Estimates of *Φ_ST_* and *D_XY_* appear above and below the diagonal, respectively.

|  | DJ | GJ | BE | JC | PC | JS | SC | HC | JA | JE | GY |
| --- | --- | --- | --- | --- | --- | --- | --- | --- | --- | --- | --- |
| DJ |  | 0.596 | 0.844 | 0.870 | 0.891 | 0.891 | 0.836 | 0.954 | 0.896 | 0.925 | 0.981 |
| GJ | 0.00385 |  | 0.484 | 0.734 | 0.576 | 0.576 | 0.492 | 0.888 | 0.756 | 0.860 | 0.957 |
| BE | 0.00443 | 0.00292 |  | 0.878 | 0.828 | 0.828 | 0.728 | 0.973 | 0.893 | 0.947 | 0.987 |
| JC | 0.00542 | 0.00558 | 0.00476 |  | 0.930 | 0.930 | 0.883 | 0.966 | 0.938 | 0.919 | 0.985 |
| PC | 0.00458 | 0.00300 | 0.00205 | 0.00549 |  | 0.000^NS^ | 0.146^NS^ | 0.989 | 0.942 | 0.965 | 0.993 |
| JS | 0.00458 | 0.00300 | 0.00205 | 0.00550 | 0.00017 |  | 0.146^NS^ | 0.989 | 0.942 | 0.965 | 0.993 |
| SC | 0.00481 | 0.00333 | 0.00241 | 0.00573 | 0.00052 | 0.00052 |  | 0.969^**^ | 0.900 | 0.941 | 0.985 |
| HC | 0.01130 | 0.01230 | 0.01280 | 0.01147 | 0.01287 | 0.01280 | 0.01311 |  | 0.969 | 0.963 | 0.993 |
| JA | 0.00617 | 0.00583 | 0.00484 | 0.00867 | 0.00558 | 0.00558 | 0.00594 | 0.01005 |  | 0.914 | 0.987 |
| JE | 0.00885 | 0.00997 | 0.01012 | 0.00693 | 0.01021 | 0.01021 | 0.01042 | 0.00965 | 0.00587 |  | 0.985 |
| GY | 0.02866 | 0.03000 | 0.03012 | 0.02883 | 0.02987 | 0.02987 | 0.03010 | 0.02984 | 0.02800 | 0.02701 |  |

All *Φ_ST_* values significantly deviated from zero (*p* < 0.001) except those denoted by ** (*p* < 0.01) and 'NS'.

Table S5. Information and summary statistics of the 14 microsatellite markers used in this study.

| Locus | Forward  primer | Reverse  primer | Repeat motif | Allele range | *N*_a_ | *N*e | *H*_O_ | *H*_E_ | u*H*_E_ | *F_IS_* |
| --- | --- | --- | --- | --- | --- | --- | --- | --- | --- | --- |
| K1004 | ATTCACTCCAAAAGCATGGG | TATGGGACGGAACAATGGAT | TCTT | 142–250 | 24 | 11.319 | 0.770 | 0.912 | 0.914 | 0.156 |
| K1005 | TCCTCACGGGCAGATGTACT | GCACCTGCTGCCTATTTCTC | GAAA | 203–259 | 15 | 9.901 | 0.799 | 0.899 | 0.901 | 0.111 |
| K1006 | ATGGCTCTCTACCTGCCAAA | GCTAGAAGCAGACGCACCTT | CTTT | 127–195 | 17 | 8.205 | 0.770 | 0.878 | 0.880 | 0.124 |
| K1008 | TGCTCTGTTGGAAGTATTGACAG | ATGATAGCCATTGTGTGCCA | TAGA | 159–227 | 18 | 10.451 | 0.824 | 0.904 | 0.907 | 0.089 |
| K1011 | AAAGAAAGCAGGCAAGAGGG | TTCTCACCTTTCGGGTTTTG | GAAA | 156–232 | 17 | 6.291 | 0.683 | 0.841 | 0.843 | 0.187 |
| K1012 | TTCCCCAGACCTAGTGGTTG | TTAGCGGTCAAGACACAACG | AGAA | 230–290 | 16 | 10.359 | 0.755 | 0.903 | 0.906 | 0.164 |
| K1018 | TAGCAGTTTAAAGCGCCTGC | CTCTGTCTGTCCTTAGCGCC | TCTA | 170–278 | 26 | 8.271 | 0.632 | 0.879 | 0.881 | 0.281 |
| K1019 | TCCTAGATTTGGGACACATGC | GGAGGACAATGCCATGAAGT | AAGA | 119–227 | 17 | 8.746 | 0.598 | 0.886 | 0.888 | 0.325 |
| K1020 | TTCCTCCCATTCAAATCTGC | CTTCCTGTTTGTGGTGCTGA | CTAT | 147–243 | 25 | 12.594 | 0.735 | 0.921 | 0.923 | 0.201 |
| K1021 | GGTGAGCAGTTTGCACTCAA | TTGTGTGTTCAAGGGTGCAT | ATCC | 162–222 | 16 | 7.658 | 0.667 | 0.869 | 0.872 | 0.233 |
| K1039 | ATTGAAGCCACTAGGCATGG | ATGCCATTGTGTTTGAGGGT | TAC | 114–159 | 13 | 4.168 | 0.309 | 0.760 | 0.762 | 0.594 |
| K1040 | CCACCAAGCAAGCATGAGTA | TTGACCTTTATGTTGGCGCT | TTA | 153–207 | 16 | 3.098 | 0.328 | 0.677 | 0.679 | 0.515 |
| K1049 | TCTGCATCAGGAACAGCACT | GCCCTGTACCTGCAATTGAT | AC | 134–190 | 24 | 4.704 | 0.539 | 0.787 | 0.789 | 0.315 |
| K1051 | GCAGCGCTTATCTCAAGGAC | GCATAAAGGCACTGTCAGCA | TG | 146–178 | 17 | 7.909 | 0.534 | 0.874 | 0.876 | 0.388 |

Abbreviations: number of alleles (*N*_a_), effective number of alleles (*N*_e_), observed heterozygosity (*H*_O_), expected heterozygosity (*H*_E_), unbiased expected heterozygosity (u*H*_E_) and fixation index (*F_IS_*).

**Table S6.** Results of historical demography analyses of *Karsenia koreana* based on 14 microsatellite loci. *M*-ratio of more than 0.68 and normal L-shaped allele frequency distribution of all populations imply no detectable genetic bottleneck of *Karsenia koreana* in South Korea. Population codes follow Table 1.

| Population | Garza-Williamson index (*M*-ratio) | BOTTLENECK (mode-shift indicator) |
| --- | --- | --- |
| DJ | 0.796 | L-shaped |
| GJ | 0.812 | L-shaped |
| BE | 0.768 | L-shaped |
| JC | 0.752 | L-shaped |
| PC | 0.893 | L-shaped |
| JS | 0.798 | L-shaped |
| SC | 0.704 | L-shaped |
| HC | 0.723 | L-shaped |
| JA | 0.716 | L-shaped |
| JE | 0.877 | L-shaped |
| GY | 0.804 | L-shaped |

**Table S7.** Parameter estimation of two chosen DIYABC scenarios among seven *Karsenia koreana* clusters based on microsatellite data. N = effective population size of the cluster, t = coalescent time measured in generations, q = quantiles for posterior mean value. For numerical code information of N and t of each scenario, see Figure S5.

(a) Scenario 1

| Parameter | Mean | Median | Mode | q025 | q050 | q250 | q750 | q950 | q975 |
| --- | --- | --- | --- | --- | --- | --- | --- | --- | --- |
| N1.1 | 2.00e+3 | 1.67e+3 | 1.55e+3 | 5.89e+2 | 7.03e+2 | 1.19e+3 | 2.36e+3 | 4.47e+3 | 5.77e+3 |
| N2.1 | 6.98e+3 | 7.08e+3 | 7.20e+3 | 3.68e+3 | 4.22e+3 | 5.98e+3 | 8.11e+3 | 9.39e+3 | 9.66e+3 |
| N3.1 | 8.74e+3 | 8.93e+3 | 9.28e+3 | 6.48e+3 | 7.03e+3 | 8.33e+3 | 9.36e+3 | 9.83e+3 | 9.91e+3 |
| N4.1 | 5.46e+3 | 5.36e+3 | 5.13e+3 | 2.14e+3 | 2.57e+3 | 4.13e+3 | 6.69e+3 | 8.84e+3 | 9.37e+3 |
| N5.1 | 5.03e+3 | 4.91e+3 | 5.10e+3 | 1.86e+3 | 2.26e+3 | 3.72e+3 | 6.16e+3 | 8.52e+3 | 9.15e+3 |
| N6.1 | 7.48e+3 | 7.62e+3 | 7.76e+3 | 4.40e+3 | 5.00e+3 | 6.68e+3 | 8.44e+3 | 9.50e+3 | 9.72e+3 |
| N7.1 | 4.40e+3 | 4.14e+3 | 3.34e+3 | 1.42e+3 | 1.75e+3 | 3.03e+3 | 5.50e+3 | 8.11e+3 | 8.87e+3 |
| t0.1 | 1.04e+4 | 5.38e+3 | 2.53e+3 | 1.02e+3 | 1.33e+3 | 2.94e+3 | 1.11e+4 | 3.90e+4 | 5.59e+4 |
| t1a.1 | 4.24e+3 | 2.95e+3 | 1.65e+3 | 6.41e+2 | 8.47e+2 | 1.78e+3 | 4.98e+3 | 1.16e+4 | 1.61e+4 |
| t1b.1 | 2.73e+3 | 2.36e+3 | 2.18e+3 | 7.83e+2 | 9.62e+2 | 1.66e+3 | 3.33e+3 | 5.60e+3 | 6.70e+3 |

(b) Scenario 2

| Parameter | Mean | Median | Mode | q025 | q050 | q250 | q750 | q950 | q975 |
| --- | --- | --- | --- | --- | --- | --- | --- | --- | --- |
| N1.2 | 1.71e+3 | 1.29e+3 | 1.15e+3 | 3.86e+2 | 4.67e+2 | 8.53e+2 | 1.98e+2 | 4.62e+2 | 6.37e+2 |
| N2.2 | 6.05e+3 | 6.05e+3 | 5.73e+3 | 2.77e+3 | 3.29e+3 | 4.87e+3 | 7.21e+3 | 8.98e+3 | 9.45e+3 |
| N3.2 | 8.07e+3 | 8.24e+3 | 8.38e+3 | 5.35e+3 | 5.95e+3 | 7.45e+3 | 8.87e+3 | 9.63e+3 | 9.81e+3 |
| N4.2 | 5.29e+3 | 5.18e+3 | 5.03e+3 | 2.08e+3 | 2.48e+3 | 3.94e+3 | 6.46e+3 | 8.65e+3 | 9.29e+3 |
| N5.2 | 4.92e+3 | 4.78e+3 | 4.66e+3 | 1.94e+3 | 2.34e+3 | 3.70e+3 | 5.95e+3 | 8.19e+3 | 8.97e+3 |
| N6.2 | 6.88e+3 | 6.97e+3 | 7.36e+3 | 3.60e+3 | 4.19e+3 | 5.94e+3 | 7.91e+3 | 9.30e+3 | 9.64e+3 |
| N7.2 | 4.04e+3 | 3.74e+3 | 2.95e+3 | 1.22e+3 | 1.51e+3 | 2.67e+3 | 5.07e+3 | 7.89e+3 | 8.78e+3 |
| t1.2 | 1.56e+4 | 1.32e+4 | 1.07e+4 | 5.15e+3 | 6.05e+3 | 9.78e+3 | 1.82e+4 | 3.22e+4 | 4.28e+4 |
| t2a.2 | 5.72e+3 | 5.71e+3 | 5.42e+3 | 1.90e+3 | 2.40e+3 | 4.31e+3 | 7.14e+3 | 9.14e+3 | 9.51e+3 |
| t2b.2 | 2.63e+3 | 2.09e+3 | 1.19e+3 | 5.39e+2 | 6.89e+2 | 1.34e+3 | 3.31e+3 | 6.82e+3 | 8.07e+3 |
| t3.2 | 3.34e+2 | 2.63e+2 | 1.92e+2 | 6.08e+1 | 8.24e+1 | 1.68e+2 | 4.00e+2 | 8.03e+2 | 1.06e+3 |

**Table S8.** Confidence evaluation of two chosen DIYABC scenarios among seven *Karsenia koreana* clusters based on microsatellite data. Numerical values in each cell indicate the number of times the scenario on the left has the highest posterior probability among 1,000 pseudo-observed data sets simulated with Scenario 1 or Scenario 2. The number of times each scenario has the highest posterior probabilities were evaluated by the direct number of the scenario selected (Direct approach), and the logistic regression estimate of the scenario selected (Logistic regression).

|  | | Pseudo-observed data sets simulated | |
| --- | --- | --- | --- |
|  |  | Scenario 1 | Scenario 2 |
| Direct approach | Scenario 1 | 328 | 60 |
|  | Scenario 2 | 672 | 940 |
| Logistic regression | Scenario 1 | 327 | 18 |
|  | Scenario 2 | 673 | 982 |


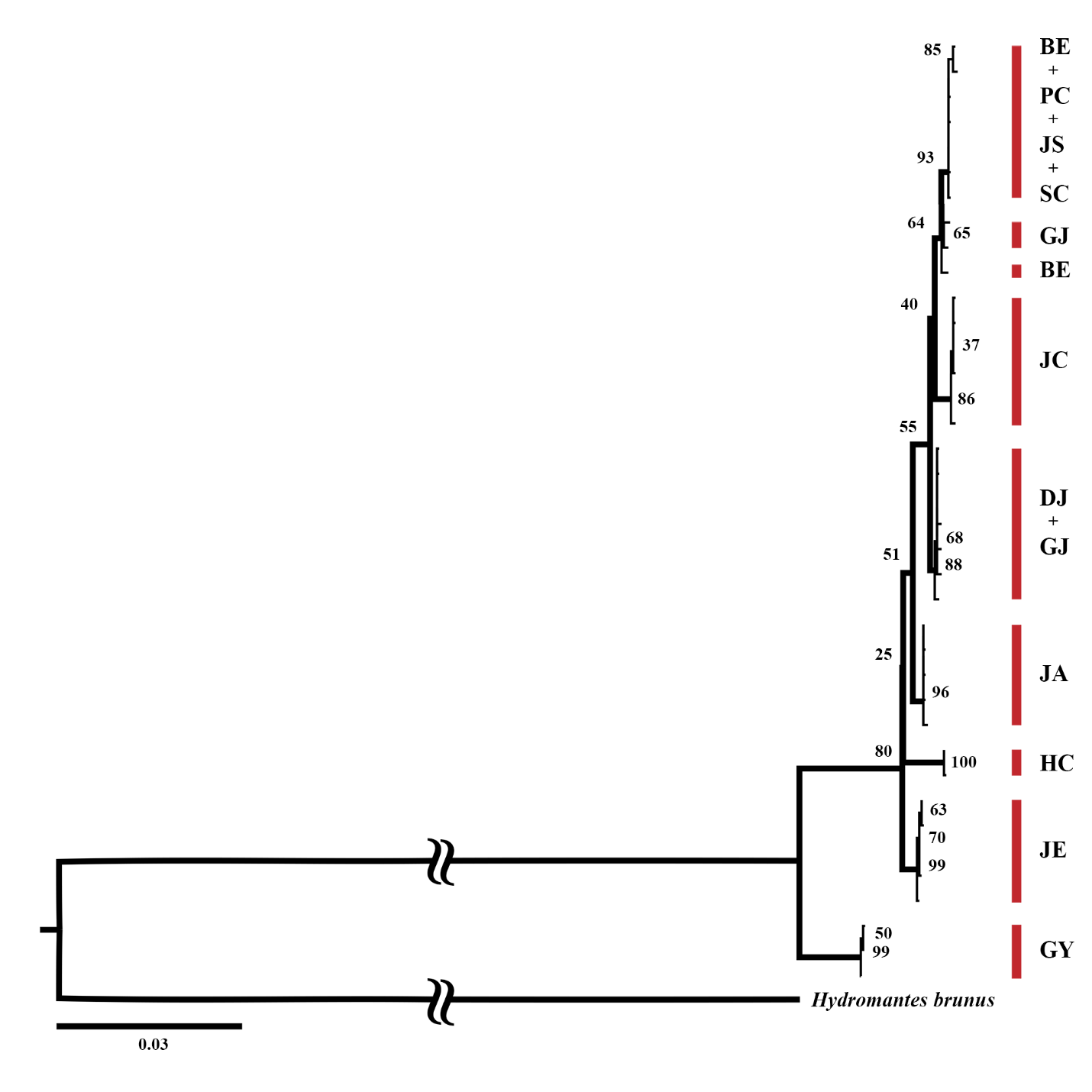


**Figure S1.** Maximum likelihood (ML) tree of *Karsenia koreana* populations reconstructed based on the concatenated sequences of cytochrome *c* oxidase I and cytochrome *b* using RaxML. Bootstrap support values are indicated at nodes. Branches within individual clusters were thinned for the visibility. Population codes follow Table 1.


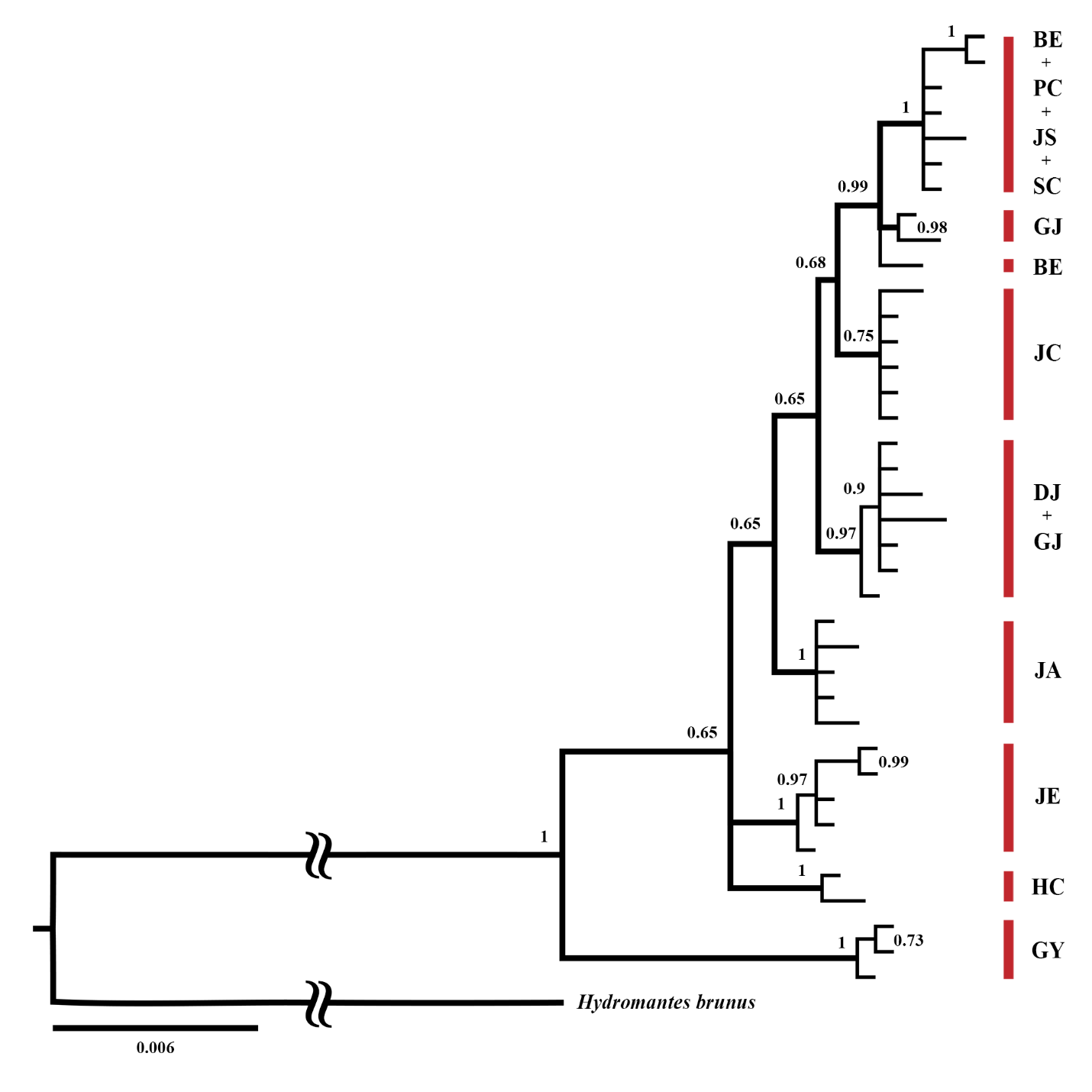


**Figure S2.** Bayesian inference (BI) tree of *Karsenia koreana* populations reconstructed based on the concatenated sequences of cytochrome *c* oxidase I and cytochrome *b* using MrBayes. Posterior probabilities are indicated at nodes. Branches within individual clusters were thinned for the visibility. Population codes follow Table 1.


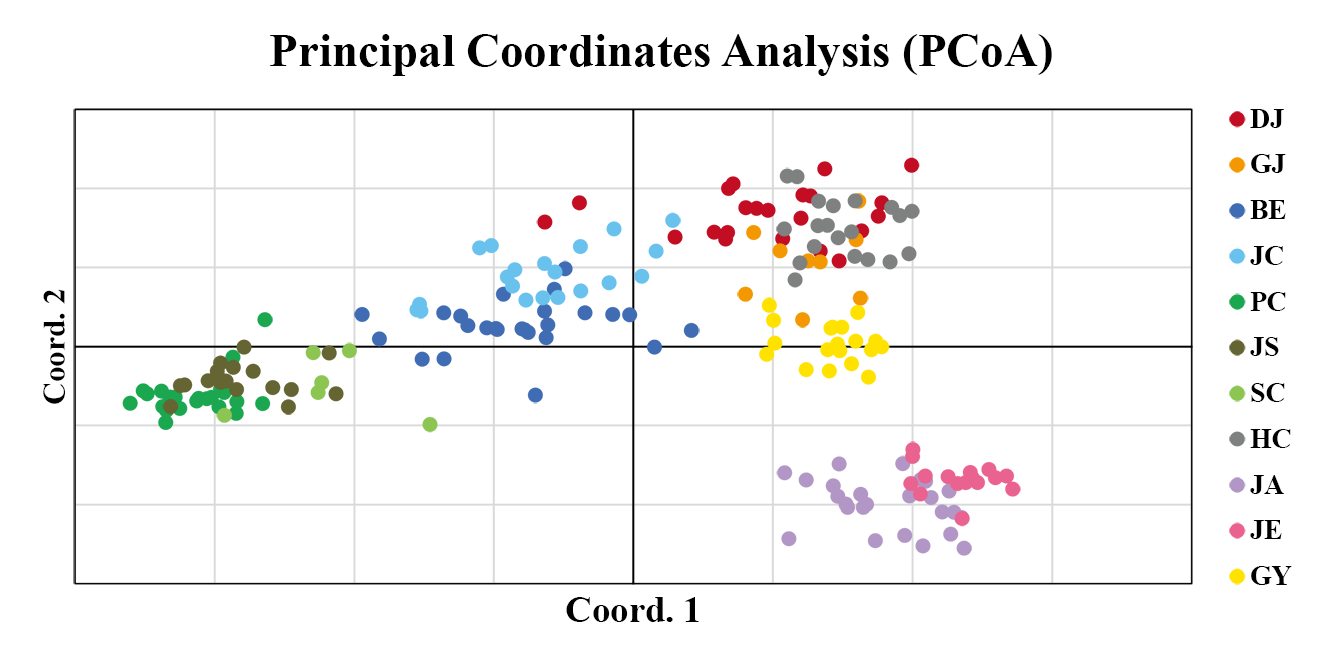


**Figure S3.** Covariance-standardized PCoA plot showing genetic differentiation based on microsatellite *F*_ST_ values among *Karsenia koreana* populations. Coordinate 1 (Coord. 1) and 2 (Coord. 2) account for 14% and 5.79 % of the total variation, respectively. Population codes follow Table 1.


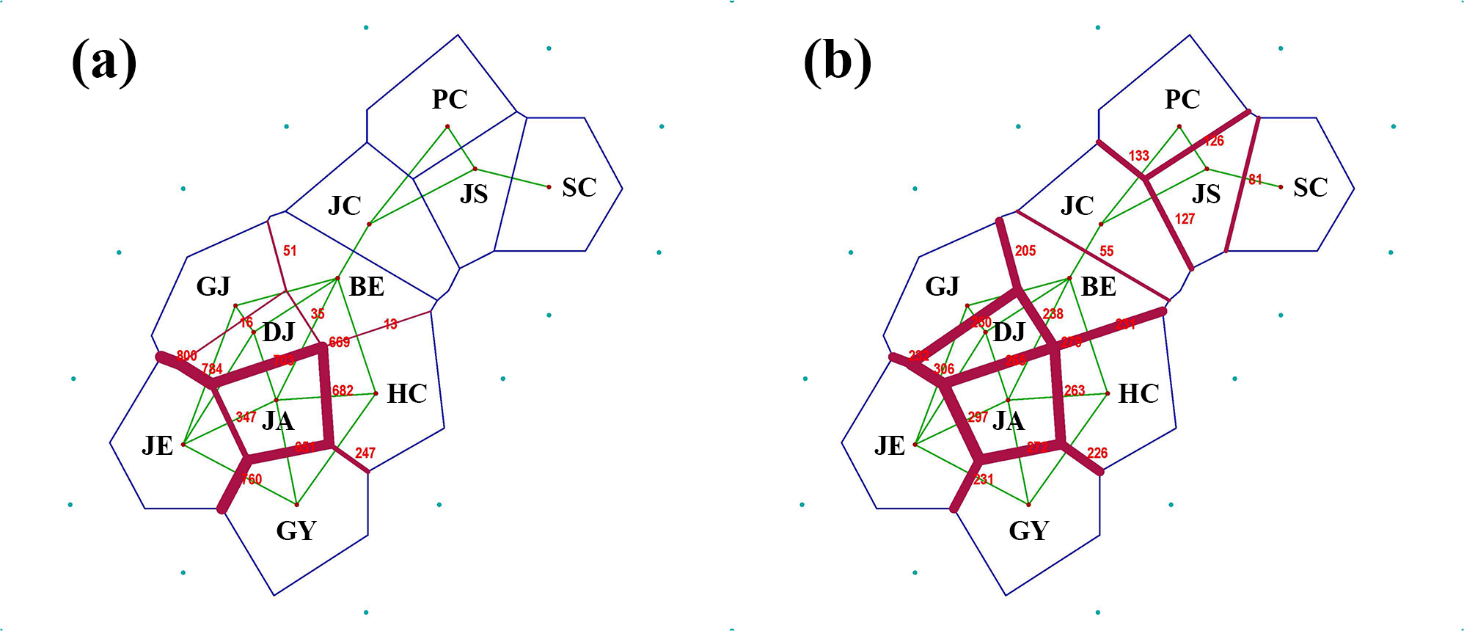


Figure S4. The results of the BARRIER analysis for *Karsenia koreana* populations estimated based on (a) bootstrapped *D_A_*, and (b) bootstrapped *F_ST_*. The bootstrap significance levels are indicated by the thickness of red-lined barriers. (a) The most conspicuous genetic barrier was inferred between group JA + JE and the other groups, between JA and JE and between HC and GY. (b) The genetic barriers are quite broad compared to (a). Population codes follow Table 1.

**
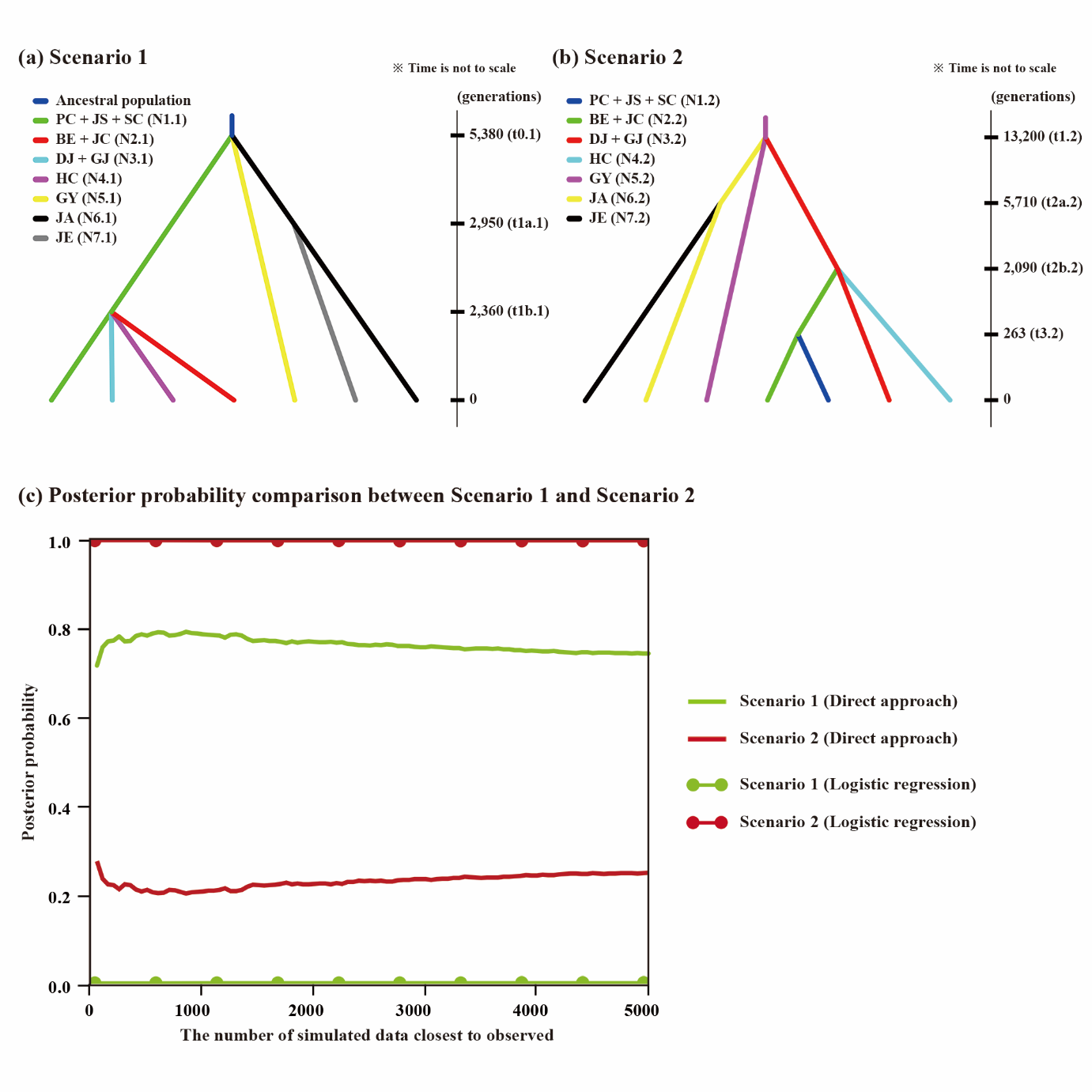
**

**Figure S5.** The products of Approximate Bayesian Computation (ABC) analysis implemented in DIYABC to identify the most likely pattern of historical divergence among populations. (a) Scenario 1. (b) Scenario 2. Divergence times on the right axis are represented in generations and not proportional to the actual scale. (c) The most likely scenario with the highest posterior probability was chosen by performing a weighted logistic regression for the probability of each scenario to compare the deviations between simulated and observed summary statistics. The single most likely scenario was estimated to be scenario 2. For confidence evaluation results between the two scenarios, see Table S7. Population codes follow Table 1.
